# Efficacy and safety of fruquintinib in patients with refractory metastatic colorectal cancer: a FRESCO-2 subgroup analysis of patients enrolled in Japan

**DOI:** 10.1007/s10147-025-02852-9

**Published:** 2025-09-01

**Authors:** Daisuke Kotani, Takayuki Yoshino, Toshiki Masuishi, Yu Sunakawa, Atsuo Takashima, Kentaro Yamazaki, Hisato Kawakami, Tomohiro Nishina, Yoshito Komatsu, Taito Esaki, Cathy Eng, Stacey Ukrainskyj, Rajash Pallai, Shivani Nanda, Zhao Yang, William Schelman, Marek Kania, Taroh Satoh

**Affiliations:** 1 Department of Gastrointestinal Oncology, National Cancer Center Hospital East, Kashiwa, Chiba Japan; 2 Department of Clinical Oncology, Aichi Cancer Center, Nagoya, Japan; 3 Department of Clinical Oncology, St Marianna University School of Medicine, Kanagawa, Japan; 4 Department of Gastrointestinal Medical Oncology, National Cancer Center Hospital, Tokyo, Japan; 5 Division of Gastrointestinal Oncology, Shizuoka Cancer Center, Shizuoka, Japan; 6 Department of Medical Oncology, Kindai University Faculty of Medicine, Osakasayama, Japan; 7 Department of Clinical Oncology, Tohoku University Graduate School of Medicine, Sendai, Japan; 8 Department of Gastrointestinal Medical Oncology, National Hospital Organization Shikoku Cancer Center, Matsuyama, Ehime, Japan; 9 Department of Cancer Chemotherapy, Hokkaido University Hospital, Cancer Center, Hokkaido, Japan; 10 Department of Gastrointestinal and Medical Oncology, NHO Kyushu Cancer Center, Fukuoka, Japan; 11 Department of Medicine, Division of Hematology and Oncology, Vanderbilt-Ingram Cancer Center, Nashville, TN USA; 12 HUTCHMED International Corporation, Florham Park, NJ USA; 13 Mural Oncology, Waltham, MA USA; 14 Department of Gastroenterological Surgery, Graduate School of Medicine, Osaka University, Osaka, Japan

**Keywords:** Fruquintinib, Japan subgroup analysis, Metastatic colorectal cancer, VEGFR inhibitor

## Abstract

**Background:**

In the phase 3 FRESCO-2 study, fruquintinib plus best supportive care (BSC) significantly improved overall survival (OS) versus placebo plus BSC in patients with refractory metastatic colorectal cancer (mCRC). We present the results of a FRESCO-2 post hoc subgroup analysis evaluating outcomes of patients enrolled in Japan.

**Methods:**

In FRESCO-2, patients had previously received all standard chemotherapies, anti-VEGF and anti-EGFR therapies if indicated, and had progressed on, or were intolerant to trifluridine-tipiracil and/or regorafenib. Patients were randomized 2:1 to receive fruquintinib 5 mg or matching placebo by mouth once daily on days 1–21 in 28-day cycles, plus BSC. The primary endpoint was OS; secondary endpoints included progression-free survival (PFS) and safety.

**Results:**

Of the 56 patients enrolled in Japan, 40 (71.4%) and 16 (28.6%) were randomized to fruquintinib and placebo, respectively. OS was improved with fruquintinib versus placebo (median 6.9 vs. 5.6 months; hazard ratio [HR], 0.42; 95% confidence interval [CI] 0.19 − 0.92). PFS was also improved with fruquintinib versus placebo (median 3.6 vs. 1.8 months; HR, 0.27; 95% CI 0.13 − 0.56). The incidence of grade ≥ 3 treatment-emergent adverse events (TEAEs) with fruquintinib versus placebo was 71.8% versus 29.4%; the most common grade ≥ 3 TEAEs with fruquintinib were hypertension (23.1%) and palmar-plantar erythrodysesthesia (17.9%).

**Conclusions:**

Fruquintinib improved OS and PFS versus placebo in FRESCO-2 patients enrolled in Japan and demonstrated a manageable safety profile. Results from the Japan subgroup were consistent with the global FRESCO-2 population, thus supporting fruquintinib as a novel treatment option for patients in Japan with refractory mCRC.

**Clinical trial details:**

ClinicalTrials.gov; NCT04322539.

**Supplementary Information:**

The online version contains supplementary material available at 10.1007/s10147-025-02852-9.

## Introduction

In Japan, colorectal cancer (CRC) was the second most diagnosed cancer in 2023, with an estimated 161,100 new cases [1]. Among females, CRC was the leading cause of cancer-related mortality, accounting for 15% of all cancer-related deaths; among males, it was the second leading cause, accounting for 13% of all cancer-related deaths [1].

Globally, around 20% of patients with CRC are diagnosed at the metastatic stage, and it is estimated that up to 50% of patients diagnosed at an earlier stage will eventually develop metastases [2]. The prognosis for patients with metastatic CRC (mCRC) remains poor, with a 5-year relative survival rate of approximately 14% [3]. Prolonging patient survival whilst maintaining quality of life are key treatment goals for patients with refractory mCRC [4].

For patients with advanced or recurrent mCRC that is refractory to standard therapies, current treatment options approved in Japan include trifluridine-tipiracil (TAS-102) [5] with or without bevacizumab [6], and regorafenib [7]. In clinical trials, both TAS-102 and regorafenib showed only incremental improvements on median overall survival (OS) compared with placebo plus best supportive care (BSC) [8, 9]. Additionally, these therapies may not be suitable for some patients due to associated toxicities; regorafenib is associated with an increased risk of hepatoxicity and palmar-plantar erythrodysesthesia (PPE) [8, 10–12], and TAS-102 is associated with high rates of myelosuppression [9, 13, 14]. The recent SUNLIGHT trial demonstrated that the addition of bevacizumab to TAS-102 improved OS versus TAS-102 alone among patients with refractory mCRC [15] and this regimen is commonly used in clinical practice in Japan [16]. Nevertheless, for pre-treated patients who are intolerant to or who progress on TAS-102 and/or regorafenib, limited treatment options are available.

The vascular endothelial growth factor (VEGF) pathway is a key mediator of angiogenesis, which is necessary for tumor growth and metastasis [17]. Fruquintinib is a selective, oral tyrosine kinase inhibitor of all three VEGF receptors (VEGFR-1, −2, and −3) [18], which restricts tumor growth and progression through inhibition of angiogenesis, with limited off-target kinase activity [18, 19]. Fruquintinib was approved in China in September 2018 as third or later line of therapy for mCRC [20], based on the results of the phase 3 FRESCO study (NCT02314819) conducted in China [21]. FRESCO met its primary endpoint by demonstrating a significant OS benefit with fruquintinib plus BSC versus placebo plus BSC (median 9.3 vs. 6.6 months; hazard ratio [HR], 0.65; 95% confidence interval [CI] 0.51 − 0.83; *P* < 0.001) [21]. The subsequent, phase 3 FRESCO-2 study (NCT04322539) evaluated the efficacy and safety of fruquintinib plus BSC in a global patient population. In FRESCO-2 (unlike in FRESCO), patients had received all standard cytotoxic and targeted therapies and had progressed on, or were intolerant to TAS-102 and/or regorafenib, reflective of current global treatment practices [22]. FRESCO-2 also met its primary endpoint by demonstrating an improvement in median OS of 2.6 months with fruquintinib plus BSC versus placebo plus BSC (7.4 months vs. 4.8 months; HR, 0.66; 95% CI 0.55 − 0.80; *P* < 0.001) [22].

Based on the results from FRESCO and FRESCO-2, fruquintinib was approved by the United States Food and Drug Administration in November 2023 for the treatment of adult patients with mCRC who have been previously treated with fluoropyrimidine-, oxaliplatin-, and irinotecan-based chemotherapy, anti-VEGF therapy, and, if *RAS* wild-type and medically appropriate, anti-epidermal growth factor receptor (EGFR) therapy [23]. In June 2024, fruquintinib was approved in the European Union for the treatment of adult patients with mCRC who have been previously treated with available standard therapies, including fluoropyrimidine, oxaliplatin-, and irinotecan-based chemotherapies, anti-VEGF agents, and anti-EGFR agents, and who have progressed on or are intolerant to treatment with either TAS-102 or regorafenib [24]. Fruquintinib was approved in Japan in September 2024 for the treatment of advanced or recurrent CRC that is neither curable nor resectable and that has progressed after chemotherapy [25].

Disparities in prognosis among patients with CRC of different ethnic origins have previously been reported [26]. Additionally, prior studies of kinase inhibitors have shown differing adverse event profiles among Japanese and non-Japanese patients [27], thus highlighting the importance of assessing the consistency of fruquintinib treatment effects in different patient populations. Therefore, we conducted a post hoc subgroup analysis of patients from FRESCO-2 who were enrolled in Japan.

## Patients and methods

### Study design and patient eligibility

Full FRESCO-2 methods have been published previously [22]. FRESCO-2 was a global, randomized, double-blind, placebo-controlled phase 3 study conducted at 124 sites across 14 countries in North America, Europe, Asia and Australia. Patients aged ≥ 18 years (≥ 20 years in Japan) with histologically/cytologically confirmed metastatic colorectal adenocarcinoma were eligible for enrollment. Eligible patients had received all standard treatments, including fluoropyrimidine, oxaliplatin, and irinotecan systemic chemotherapy, anti-VEGF therapy and anti-EGFR therapy (if *RAS* wild-type); had progressed on or were intolerant to TAS-102 and/or regorafenib; and had received an immune checkpoint inhibitor or BRAF inhibitor therapy if indicated. For this post hoc Japan subgroup analysis, patients enrolled in Japan in both the fruquintinib, and placebo arms of FRESCO-2 were included. This analysis is descriptive only.

### Randomization and treatment

Patients were randomized 2:1 to receive oral fruquintinib 5 mg or matching placebo once daily in 28-day cycles, 3 weeks on, 1 week off, plus BSC, until treatment progression or unacceptable toxicity. Randomization was stratified by prior therapy (TAS-102 vs. regorafenib vs. both TAS-102 and regorafenib); *RAS* mutational status (wild-type vs. mutant), and duration of metastatic disease (≤ 18 vs. > 18 months) [22].

### Endpoints

The primary endpoint of FRESCO-2 was OS, defined as the time from randomization to death from any cause. Secondary endpoints included progression-free survival (PFS), objective response rate, disease control rate (DCR), and safety, which was monitored by an independent data monitoring committee.

FRESCO-2 was conducted in accordance with the Declaration of Helsinki and Good Clinical Practice guidelines, including the International Council for Harmonisation of Technical Requirements for Pharmaceuticals for Human Use, and all applicable laws and regulations. The protocol was approved by the institutional review boards and independent ethics committees at each site. All participating patients provided written informed consent [22].

### Statistical analysis

Statistical methods for FRESCO-2 have been previously published [22]. Median OS and PFS were estimated via Kaplan–Meier analysis with 95% CIs calculated from a log–log transformation based on the Brookmeyer–Crowley method. Adjusted HRs and 95% CIs were estimated using the Cox proportional hazards model, using the randomization schedule stratification factors and treatment group as covariates. *P*-values were calculated using the stratified log-rank test. These analyses were not prespecified, and the study was not powered to test for statistical significance—all statistics are descriptive only.

## Results

### Patients

**Fig. 1:**
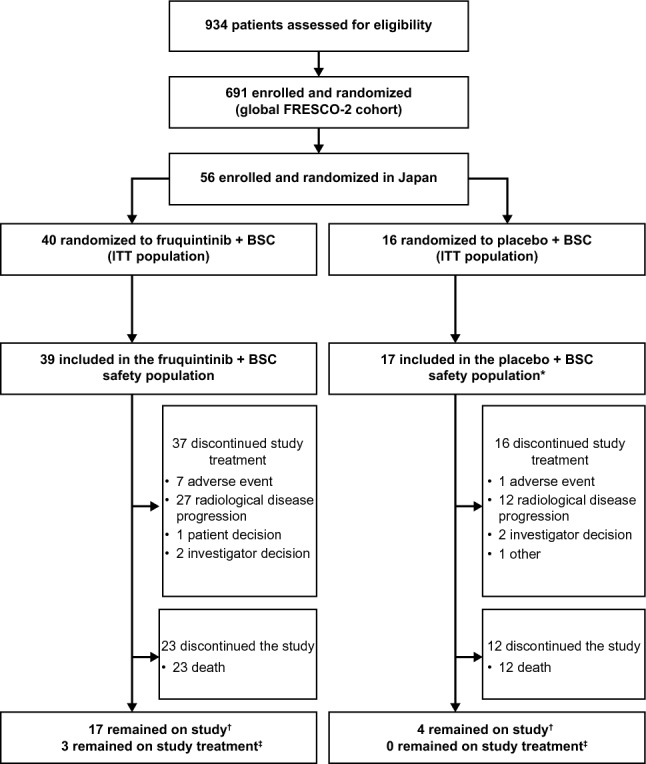
Patient disposition. *One patient randomized to the fruquintinib arm received placebo instead. ^†^Patients with missing end-of-study information were considered to be remaining on study. ^‡^Patients who received study drug but had missing end-of-treatment information were considered to be remaining on treatment. BSC, best supportive care; ITT, intention-to-treat

Between April 20, 2021, and November 08, 2021, 56 patients were enrolled from 10 sites across Japan and randomized to fruquintinib plus BSC (*n* = 40) or placebo plus BSC (*n* = 16). As of data cut-off (June 24, 2022), three patients in the fruquintinib arm and no patients in the placebo arm remained on treatment (Fig. 1). Baseline demographics are shown in Table 1. All patients in the fruquintinib arm were Asian; 15 patients (93.8%) in the placebo arm were Asian and one patient was Black/African American. Among patients in the fruquintinib and placebo arms, 65.0% and 50.0%, respectively, had an Eastern Cooperative Oncology Group performance status (ECOG PS) of 0, and 35.0% and 25.0% had no liver metastases at baseline. Overall, 85.0% of patients in the fruquintinib arm and all patients in the placebo arm had a duration of metastatic disease > 18 months. Patients had received a median of 5.0 and 5.5 prior lines of therapy in the fruquintinib and the placebo arms, respectively. In total, 75.0% of patients in the fruquintinib arm and 87.5% of patients in the placebo arm had received > 3 prior treatment lines for metastatic disease. Overall, 100% and 25.0% of patients in the fruquintinib arm had received prior VEGF and EGFR inhibitors, respectively; of patients in the placebo arm, 100% and 43.8% had received prior VEGF and EGFR inhibitors, respectively. Among patients in the fruquintinib and placebo arms, 75.0% and 68.8%, respectively, had received prior treatment with both TAS-102 and regorafenib; 22.5% and 25.0% had received prior TAS-102 but not regorafenib; and 2.5% and 6.3% had received prior regorafenib but not TAS-102 (Table 1).

**Table 1:** Baseline demographics and disease characteristics of FRESCO-2 patients enrolled in Japan (ITT population*)

Characteristic	Fruquintinib + BSC (*n* = 40)	Placebo + BSC (*n* = 16)
Age, years		
Median (range)	61.0 (47 − 78)	59.5 (30 − 76)
Aged ≥ 65 years (%)	15 (37.5)	5 (31.3)
Male, *n* (%)	18 (45.0)	9 (56.3)
Race, *n* (%)		
Asian	40 (100)	15 (93.8)
Black/African American	0	1 (6.3)
ECOG PS, *n* (%)		
0	26 (65.0)	8 (50.0)
1	14 (35.0)	8 (50.0)
Primary site at first diagnosis, *n* (%)		
Colon, left	14 (35.0)	8 (50.0)
Colon, right	9 (22.5)	2 (12.5)
Rectum	17 (42.5)	6 (37.5)
Liver metastases, *n* (%)		
Yes	26 (65.0)	12 (75.0)
No	14 (35.0)	4 (25.0)
Duration of metastatic disease, months, *n* (%)		
≤ 18	6 (15.0)	0
> 18	34 (85.0)	16 (100)
*RAS* status, *n* (%)		
Wild type	9 (22.5)	7 (43.8)
Mutant	31 (77.5)	9 (56.3)
*BRAFV600E* mutation, *n* (%)		
No	36 (90.0)	13 (81.3)
Yes	1 (2.5)	0
Unknown/other	3 (7.5)	3 (18.8)
Microsatellite or mismatch repair, *n* (%)		
MSS or pMMR	39 (97.5)	15 (93.8)
MSI-H or dMMR	0	0
Unknown	1 (2.5)	1 (6.3)
Number of prior lines of treatment, median (range)	5.0 (3 − 14)	5.5 (4 − 10)
Number of prior lines of treatment in metastatic disease, *n* (%)		
≤ 3	10 (25.0)	2 (12.5)
> 3	30 (75.0)	14 (87.5)
Prior therapies, *n* (%)		
VEGF inhibitor	40 (100)	16 (100)
EGFR inhibitor	10 (25.0)	7 (43.8)
Immune checkpoint inhibitor	0	0
BRAF inhibitor	1 (2.5)	1 (6.3)
Prior TAS-102 or regorafenib, *n* (%)		
TAS-102	9 (22.5)	4 (25.0)
Regorafenib	1 (2.5)	1 (6.3)
Both	30 (75.0)	11 (68.8)

^*^Percentages are based on the number of patients in each subgroup, as defined in each column header

BSC, best supportive care; dMMR, deficient mismatch repair; ECOG PS, Eastern Cooperative Oncology Group performance status; EGFR, epidermal growth factor receptor; ITT, intention-to-treat; MSI-H, high microsatellite instability; MSS, microsatellite stable; pMMR, proficient mismatch repair; VEGF, vascular endothelial growth factor

### Efficacy

**Fig. 2:**
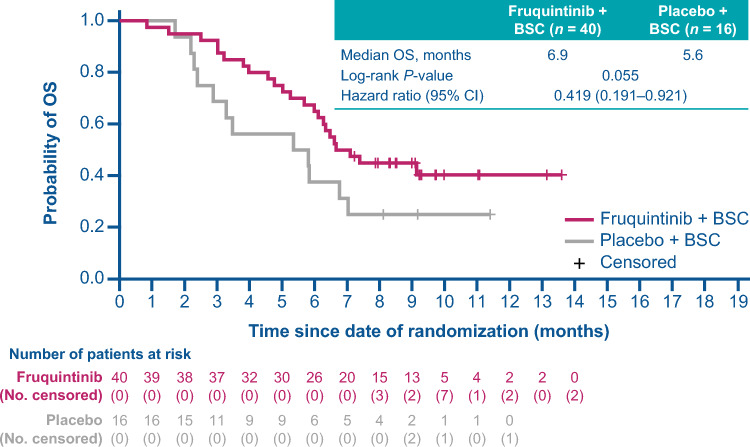
OS from the date of randomization (ITT population). BSC, best supportive care; CI, confidence interval; HR, hazard ratio; ITT, intention-to-treat; OS, overall survival

**Fig. 3:**
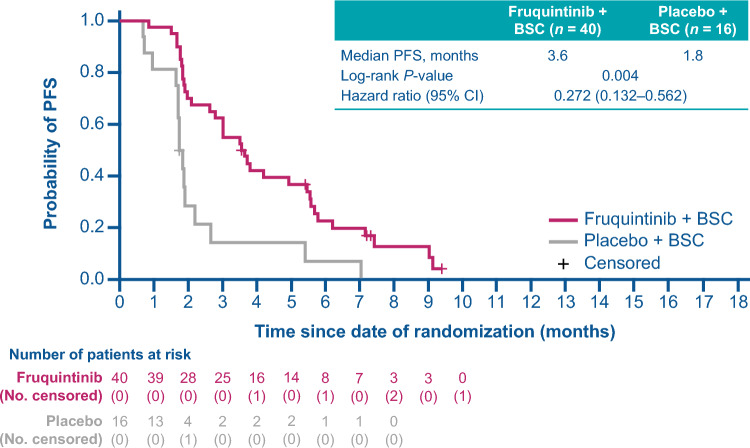
PFS from the date of randomization (ITT population). BSC, best supportive care; CI, confidence interval; HR, hazard ratio; ITT, intention-to-treat; PFS, progression-free survival

For patients in Japan receiving fruquintinib versus placebo, the median duration of follow-up was 9.2 versus 8.6 months. Median OS was improved for patients receiving fruquintinib versus placebo with a median of 6.9 versus 5.6 months (HR, 0.42; 95% CI 0.19 − 0.92; *P* = 0.055; Fig. 2). The proportion of patients receiving fruquintinib who had disease progression or died was 87.5% versus 93.8% for placebo. PFS was improved for patients receiving fruquintinib versus placebo (median 3.6 versus 1.8 months; HR, 0.27; 95% CI 0.13 − 0.56; *P* = 0.004; Fig. 3). One patient in the fruquintinib arm (2.5%) had a partial response and no patients had a complete response; there were no responses recorded in the placebo arm. The DCR was 62.5% with fruquintinib versus 25.0% for placebo (adjusted difference: 55.4%; 95% CI 27.9 − 82.9; *P* = 0.001; Table 2).

**Table 2:** Summary of response

Category	Fruquintinib + BSC (*n* = 40)	Placebo + BSC (*n* = 16)
Best objective response, *n* (%)		
Complete response	0	0
Partial response	1 (2.5)	0
Stable disease	24 (60.0)	4 (25.0)
Progressive disease	12 (30.0)	10 (62.5)
NA	3 (7.5)	2 (12.5)
Confirmed ORR (CR + PR)	1 (2.5)	0
Adjusted difference (95% CI)	2.9 (− 3.1, 8.9)	
Two-sided *P*-value	0.564	
DCR (CR + PR + SD)	25 (62.5)	4 (25.0)
Adjusted difference (95% CI)	55.4 (27.9 − 82.9)	
Two-sided *P*-value	0.001	

BSC, best supportive care; CI, confidence interval; CR, complete response; DCR, disease control rate; NA, not available; ORR, objective response rate; PR, partial response; SD, stable disease

During follow-up, 30.8% versus 41.2% of patients received ≥ 1 subsequent anti-cancer medication following fruquintinib versus placebo. The most common subsequent therapies in the fruquintinib arm were regorafenib (12.8%), bevacizumab (10.3%), and oxaliplatin (10.3%); the most common subsequent therapies in the placebo arm were regorafenib (17.6%) and fluorouracil (11.8%).

### Safety

Safety was assessed in all patients who had received at least one dose of fruquintinib or placebo. The safety population comprised 39 patients in the fruquintinib arm and 17 patients in the placebo arm (one patient who was randomized to fruquintinib received placebo instead). The median duration of exposure was 3.6 months in the fruquintinib arm versus 1.8 months in the placebo arm, with a median of 4.0 versus 2.0 treatment cycles received (Supplementary Table S1). Median relative dose intensity for fruquintinib versus placebo was 87.5% versus 99.4%. In the fruquintinib versus placebo arms, 48.7% versus 5.9% of patients had a least 1 dose reduction from 5 to 4 mg; seven patients (17.9%) in the fruquintinib arm had a further dose reduction from 4 to 3 mg. No patients required more than 2 dose reductions (Supplementary Table S1).

Among patients who received fruquintinib versus placebo, 97.4% versus 82.4% had at least one treatment-emergent adverse event (TEAE), with 71.8% versus 29.4% experiencing a grade ≥ 3 TEAE (Table 3). In the fruquintinib arm, the most common TEAEs were hypertension (any grade: 53.8%; grade ≥ 3: 23.1%), proteinuria (any grade: 51.3%; grade ≥ 3: 7.7%), and PPE (any grade: 43.6%; grade ≥ 3: 17.9%; Table 4). There were no reports of the myelotoxicity indicators neutropenia or leukopenia in either treatment arm; one patient (2.6%) who received fruquintinib had thrombocytopenia. TEAEs led to dose interruption, reduction, or discontinuation in 51.3%, 48.7%, and 15.4% of the 39 patients who received fruquintinib, and 11.8%, 5.9% and 5.9% of the 17 patients who received placebo (Table 3). TEAEs leading to dose reduction and discontinuation are detailed in Supplementary Table S2. Four (10.3%) and three (17.6%) patients in the fruquintinib and placebo arms, respectively, died due to TEAEs, none of which were considered related to treatment (Table 3).

**Table 3:** Safety summary (safety population*)

*n* (%)	Fruquintinib + BSC (*n* = 39)	Placebo + BSC (*n* = 17)
Any TEAE	38 (97.4)	14 (82.4)
Any-grade treatment-related	38 (97.4)	8 (47.1)
Grade ≥ 3	28 (71.8)	5 (29.4)
Grade ≥ 3 treatment-related	23 (59.0)	1 (5.9)
Any serious TEAE	16 (41.0)	3 (17.6)
Grade ≥ 3	15 (38.5)	3 (17.6)
TEAE leading to dose modification		
Interruption	20 (51.3)	2 (11.8)
Reduction	19 (48.7)	1 (5.9)
Discontinuation	6 (15.4)	1 (5.9)
TEAE leading to death^†^	4 (10.3)	3 (17.6)

^*^One patient randomized to the fruquintinib arm received placebo instead; ^†^TEAEs leading to death in the fruquintinib arm were disease progression (*n* = 3) and pneumonia (*n* = 1); TEAEs leading to death in the placebo arm were disease progression (*n* = 2) and malignant neoplasm progression (*n* = 1). None of the TEAEs leading to death were considered treatment related

BSC, best supportive care; TEAE, treatment-emergent adverse event

**Table 4:** TEAEs of any grade occurring in > 10% of patients (safety population*)

	Fruquintinib + BSC (*n* = 39)		Placebo + BSC (*n* = 17)		
*n* (%)	Any grade	Grade ≥ 3	Any grade		Grade ≥ 3
Hypertension	21 (53.8)	9 (23.1)	1 (5.9)		0
Proteinuria	20 (51.3)	3 (7.7)	1 (5.9)		0
PPE	17 (43.6)	7 (17.9)	3 (17.6)		0
Hypothyroidism	11 (28.2)	1 (2.6)	0		0
Stomatitis	8 (20.5)	1 (2.6)	2 (11.8)		0
Constipation	7 (17.9)	0	0		0
Platelet count decreased	7 (17.9)	0	0		0
Diarrhea	6 (15.4)	2 (5.1)	1 (5.9)		0
Dysphonia	6 (15.4)	0	1 (5.9)		0
Malaise	5 (12.8)	1 (2.6)	2 (11.8)		0
Nausea	5 (12.8)	0	2 (11.8)		0
Decreased appetite	4 (10.3)	2 (5.1)	4 (23.5)		1 (5.9)
Pyrexia	4 (10.3)	0	1 (5.9)		0

^*^One patient randomized to the fruquintinib arm received placebo instead

BSC, best supportive care; PPE, palmar-plantar erythrodysesthesia; TEAE, treatment-emergent adverse event

## Discussion

There remains a substantial unmet need for effective treatment options for patients with refractory mCRC in Japan. Although improvements in prevention and treatment have led to a reduction in the incidence of CRC and associated mortality in Japan in recent years [28], CRC remains the second most diagnosed cancer in Japan and accounts for approximately 14% of all cancer-related deaths [29].

In this post hoc analysis of FRESCO-2, patients with refractory mCRC enrolled in Japan who received fruquintinib had a longer median OS and PFS relative to patients who received placebo (OS, 6.9 vs. 5.6 months; PFS, 3.6 vs. 1.8 months). In addition, DCR was improved with fruquintinib compared with placebo (62.5% vs. 25.0%).

There were some differences in the baseline characteristics of patients receiving fruquintinib in the Japan subgroup versus patients receiving fruquintinib in the global FRESCO-2 population [22]. Patients who received fruquintinib in the Japan subgroup were younger compared with patients who received fruquintinib in the global population (37.5% were ≥ 65 years of age vs. 46.4% in the global population), they had improved baseline performance status (65.0% had an ECOG PS of 0 at baseline vs. 42.5% in the global population), and more patients in the Japan subgroup had no liver metastases at baseline (35.0% vs. 26.5% in the global population).

Compared with 691 patients in the global FRESCO-2 population, only 56 patients (8.1%) are included in this subgroup analysis. Prior treatment patterns differed for these 56 patients compared with the global FRESCO-2 population, with a higher proportion of patients in the Japan subgroup versus the global population having previously received both regorafenib and TAS-102 (75.0% vs. 39.3%), and a lower proportion having received either regorafenib alone (2.5% versus 8.7%) or TAS-102 alone (22.5% versus 52.0%) [22]. Additionally, 96.4% and 76.8% of patients in the Japan subgroup versus 91.6% and 47.8% of patients in the global FRESCO-2 population received prior TAS-102 and regorafenib, respectively. These differences may be due to differing mCRC treatment practices in Japan versus outside of Japan, or differences in access to/reimbursement of regorafenib between countries [22, 30–33]. We also note that the follow-up period for patients in the Japan subgroup was shorter in comparison with the global population (fruquintinib: 9.2 vs. 11.3 months; placebo: 8.6 vs. 11.2 months).

Despite the differences in baseline characteristics, the smaller sample size, the differing prior treatment patterns, and the shorter median follow-up time in the Japan subgroup analysis versus the global FRESCO-2 population, the median OS, median PFS, and DCR results observed are consistent with those reported in the global FRESCO-2 study, in which significant and clinically meaningful improvements in median OS (7.4 months vs. 4.8 months; HR, 0.66; 95% CI 0.55 − 0.80; *P* < 0.001), median PFS (3.7 months vs. 1.8 months; HR, 0.32; 95% CI 0.27 − 0.39; *P* < 0.001), and DCR (56% vs. 16%; *P* < 0.001) were seen in patients who received fruquintinib versus placebo [22]. Consistency of findings with the global FRESCO-2 population are supported by a population pharmacokinetics (PK) modelling analysis that investigated the impact of interpatient variability on the PK profiles of fruquintinib and its major metabolite M11; the analysis included patient data from six fruquintinib studies, including FRESCO-2 [34]. Results demonstrated that patient baseline demographics, including race (Asian, Black, White), had no clinically meaningful impact on the PK profile of fruquintinib or M11 [34]. Altogether, these findings are supportive of fruquintinib being used as a standard therapy for pretreated mCRC in Japan, regardless of having received prior regorafenib or TAS-102.

Results are also consistent with the findings of the phase 3 FRESCO study conducted in China, which demonstrated significant and clinically meaningful improvements in median OS (9.3 months vs. 6.6 months; HR, 0.65; 95% CI 0.51–0.83; *P* < 0.001) and PFS (3.7 months vs. 1.8 months; HR, 0.26; 95% CI 0.21 − 0.34; *P* < 0.001) with fruquintinib versus placebo in patients with refractory mCRC [21]. Of note, patients who received fruquintinib in FRESCO were less pre-treated versus patients in FRESCO-2. In FRESCO, 30% of patients had received prior anti-VEGF therapy and 14% had had prior anti-EGFR therapy versus 97% and 39%, respectively, in FRESCO-2; additionally, TAS-102 was not approved for use in China at the time FRESCO was conducted and patients with prior regorafenib exposure were excluded [21].

TEAEs were manageable among FRESCO-2 patients receiving fruquintinib who were enrolled in Japan and were consistent with the global FRESCO-2 safety population and the mechanism of action of VEGFR 1–3 inhibition [22, 35]. Notably, a higher proportion of patients who received fruquintinib had grade ≥ 3 TEAEs in the Japan subgroup versus the global population (71.8% vs. 62.7%); however, similar to the global population, these were generally manageable with dose modifications. For patients in the Japan subgroup who received fruquintinib, 48.7% had a dose reduction due to TEAEs compared with 24.1% of patients receiving fruquintinib in the global population; however, only 15.4% of patients in the Japan subgroup discontinued fruquintinib due to TEAEs, compared with 20.4% in the global population. Among patients receiving fruquintinib in the Japan subgroup versus patients receiving fruquintinib in the global population, there was a higher rate of hypertension (53.8% vs. 36.8%), proteinuria (51.3% vs. 17.3%), and PPE (43.6% vs. 19.3%) reported. In the global FRESCO-2 safety population, fruquintinib-related hypertension, proteinuria, and PPE were reported to resolve rapidly with hypertension resolving in a median of 16.5 days, proteinuria in a median of 15 days and PPE in a median of 39 days [36]. Indeed, despite the higher rate of these TEAEs among patients in the Japan subgroup versus the global population, only 1/39 patients receiving fruquintinib in the Japan subgroup discontinued treatment due to proteinuria (vs. 4/456 patients in the global population) and no patients discontinued treatment due to hypertension or PPE (vs. 2/456 and 3/456 patients, respectively, in the global population). In contrast to TAS-102, which has been associated with high rates of myelosuppression [37], in the patients who received fruquintinib in the Japan subgroup, there were no cases of neutropenia or leukopenia and only one patient had thrombocytopenia.

This analysis has a few limitations which should be considered. These data are from an unplanned post hoc analysis of a subgroup of patients from the FRESCO-2 study, who were enrolled in Japan. Due to the small sample size of the subgroup (8.1% of the global FRESCO-2 population), the analysis does not have sufficient statistical power to draw definitive conclusions regarding the efficacy of fruquintinib among this subpopulation. Although the overall FRESCO-2 population was stratified, geographical region was not a stratification factor, and this subgroup analysis of patients enrolled in Japan was not stratified. Therefore, there were some differences in the baseline characteristics between the treatment arms. Furthermore, due to the small sample size, analysis of OS and PFS by different patient demographic subgroups among this population was not possible. Nevertheless, this subgroup analysis confirms the efficacy findings observed for fruquintinib in the global FRESCO-2 study and provides additional safety data for patients in Japan with mCRC.

In conclusion, in this subgroup of pretreated patients with mCRC from the FRESCO-2 study who were enrolled in Japan, fruquintinib plus BSC improved OS and PFS compared with placebo plus BSC, and fruquintinib demonstrated a manageable safety profile. Results for the Japan subgroup analysis were consistent with the global FRESCO-2 population. Approval of fruquintinib offers a valuable possibility for patients with mCRC in later line settings to receive a meaningful extension in survival whilst maintaining quality of life. Overall, these findings support fruquintinib as a new oral treatment option for patients with refractory mCRC that will enrich the continuum of care for these patients.

## Data material and/or code availability

The datasets, including the redacted study protocol, redacted statistical analysis plan, and individual participants’ data supporting the results reported in this article, will be made available from the completed study within 3 months from initial request, to researchers who provide a methodologically sound proposal. The data will be provided after its de-identification, in compliance with applicable privacy laws, data protection and requirements for consent and anonymization.

## Supplementary Information

Below is the link to the electronic supplementary material.

Supplementary file1 (DOCX 35 kb)
