# Supplementary material for: Efficacy and safety of fruquintinib in patients with refractory metastatic colorectal cancer: a FRESCO-2 subgroup analysis of patients enrolled in Japan
Source: Int J Clin Oncol. 2025 Sep 1;30(10):2043–52. doi: 10.1007/s10147-025-02852-9 (PMC12474579; doi:10.1007/s10147-025-02852-9)
Supplement: Supplementary file 1 — Supplementary file1 (DOCX 35 kb) [file 10147_2025_2852_MOESM1_ESM.docx]

**SUPPLEMENTARY MATERIAL**

Efficacy and safety of fruquintinib in patients with refractory metastatic colorectal cancer: a FRESCO-2 subgroup analysis of patients enrolled in Japan

**Authors:**
Daisuke Kotani · Takayuki Yoshino · Toshiki Masuishi · Yu Sunakawa · Atsuo Takashima · Kentaro Yamazaki · Hisato Kawakami · Tomohiro Nishina · Yoshito Komatsu · Taito Esaki · Cathy Eng · Stacey Ukrainskyj · Rajash Pallai · Shivani Nanda · Zhao Yang · William Schelman · Marek Kania · Taroh Satoh

**SUPPLEMENTARY TABLES**

**Supplementary Table 1. Treatment exposure (safety population*)**

| **Category** | **Fruquintinib + BSC  (*n* = 39)** | **Placebo + BSC  (*n* = 17)** |
| --- | --- | --- |
| **Median duration of exposure, months (IQR)** | 3.61 (1.84−5.75) | 1.84 (0.92−1.94) |
| **Number of treatment cycles received, median (IQR)** | 4 (2−6) | 2 (1−2) |
| **Relative dose intensity, median, % (IQR)** | 87.464 (70.303−98.765) | 99.408 (96.970−100.000) |
| **Patients with dose modification, *n* (%)** | 31 (79.5) | 6 (35.3) |
| **Patients with drug interruption, n (%)** | 30 (76.9) | 6 (35.3) |
| **Patients with any dose reduction, *n* (%)**  From 5 mg to 4 mg  From 4 mg to 3 mg | 19 (48.7)  19 (48.7)  7 (17.9) | 1 (5.9)  1 (5.9)  0 |
| **Frequency of dose reduction, n (%)**  0 reductions  1 reduction  2 reductions  ≥3 reductions | 20 (51.3)  12 (30.8)  7 (17.9)  0 | 16 (94.1)  1 (5.9)  0  0 |

*One patient randomized to the fruquintinib arm received placebo instead.
BSC, best supportive care; IQR, interquartile range.

**Supplementary Table 2 TEAEs leading to dose reduction or dose discontinuation (safety population*)**

|  |  | |
| --- | --- | --- |
|  | **Fruquintinib + BSC  (*n* = 39)** | **Placebo + BSC  (*n* = 17)** |
| **Total patients with TEAEs leading to dose reduction, *n* (%)** | 19 (48.7) | 1 (5.9) |
| PPE | 4 (10.3) | 0 |
| Hypertension | 3 (7.7) | 0 |
| Decreased appetite | 2 (5.1) | 0 |
| Diarrhea | 2 (5.1) | 0 |
| Bile duct stone | 1 (2.6) | 0 |
| Blood bilirubin increased | 1 (2.6) | 0 |
| Electrocardiogram QT prolonged | 1 (2.6) | 0 |
| Hematuria | 1 (2.6) | 0 |
| Hepatic function abnormal | 1 (2.6) | 0 |
| Hyperlipasemia | 1 (2.6) | 0 |
| Platelet count decreased | 1 (2.6) | 0 |
| Proteinuria | 1 (2.6) | 0 |
| Stomatitis | 1 (2.6) | 0 |
| Hyperkalemia | 0 | 1 (5.9) |
| **Total patients with TEAEs leading to dose discontinuation, *n* (%)** | 6 (15.4) | 1 (5.9) |
| Cerebral infarction | 1 (2.6) | 0 |
| Dehydration | 1 (2.6) | 0 |
| Gastrointestinal hemorrhage | 1 (2.6) | 0 |
| Posterior reversible encephalopathy syndrome | 1 (2.6) | 0 |
| Proteinuria | 1 (2.6) | 0 |
| Tumor pain | 1 (2.6) | 0 |
| Decreased appetite | 0 | 1 (5.9) |

*One patient randomized to the fruquintinib arm received placebo instead.
BSC, best supportive care; PPE, palmar-plantar erythrodysesthesia; TEAE, treatment-emergent adverse event.
